# Practical Notes and Formulæ

**Published:** 1888-10

**Authors:** 


### PRACTICAL NOTES AND FORMULÆ.

**Nervous Headaches.**—Dr. Jas. Little, in *Dublin Journal of Science*, says: Of the means which are useful in lessening the frequency of these headaches, the most efficacious are abundant exercise in the open air, a nourishing diet, sponging with hot water in the morning, followed by the cold douch over the shoulders and spine, and a very sparing use of tea. Among drugs the combination which has appeared to do most good is a pill containing:

℞. Sodii arseniat.................................gr. iss,

Ext. cannabis Ind..............................gr. ⅙,

Ext. balladonnæ.......................... .......gr. ⅓,

Zinci valerianat...............................gr. ij.

M. Sig.—Take after breakfast and dinner.

**Formula for Antifebrine.**—Dr. I. N. Love (*Weekly Review*, July 7, 1888,) says that the following formula is a pleasant and convenient form of administering antifebrine:

℞. Antifebrine.................................℈ ij,

Alcohol....................................f ʒ ij.

Glycerine..................................f ʒ iij,

Cinnamon water..............................f ℥ j,

Simple syrup.......·.......................f ʒ iij

M. Sig.—One half teaspoonful to two teaspoonfuls every two to four hours, according to age and necessities.—*Med. and Surg. Reporter.*

**Formula for Dysmenorrhœa.**—*L'Union Medicale du Canada* says Calvin's formula is—

℞. Tincture of gelsemium........................
Camphor water...............................
Deodorized tincture of opium. ............aa f ℥ ij.

M. Dose, 30 drops every two hours.—*Ex.*

**Acute Tonsilitis.**—The following gargle is prescribed in the University of Pennsylvania:

℞. Potasii bromidi...............................ʒ iv,

Potasii chloratis..............................ʒ j,

Tr. ferri chlor................................f ʒ ijss,

Ext. glycyrrhizæ........ ........................ʒ j,

Aquæ........·.........................q.s. ad f ℥ iv.

M. Sig.—A teaspoonful in water every two hours; gargle and swallow.—*Medical and Surgical Reporter.*

**For Diphtheria.**—Dr. W. T. Mason (*Medical World*,) says: In diphtheria I use whisky—plenty of it—in the shape of egg nogg, *ad libitum*; also plenty of powdered sulphur, put as nearly on the membrane as possible with a feather or the handle of a spoon. What better germicide is there than sulphur? Of course I use other remedies, but my chief reliance is upon whisky (egg nogg) and sulphur.

**Removing Indelible Ink.**—Physicians are often asked how to remove indelible ink, and they sometimes cannot quite remember; so we repeat the following method: First moisten the stain with tincture of iodine, and, after a few minutes, remove the iodine stain with a solution of hyposulphite of soda. Finally wash in clean water. Repeat if necessary.—*Med. and Sur. Reporter.*

**Napthaline in Dysentery.**—Hinterhof (*Russkaya Meditzina*) praises the good effects of naphthaline in dysentery. His formula is—

℞. Pure napthaline ............................. gr. xv,
Distilled water ............................. f ℥ ij.

To be used in two enemas.

Besides the naphthaline, he administers quinine. Tenesmus and smarting at the anus subside at the first enema, and recovery is established after the second.—*Bulletin Medical.*

**Hypodermic Injections in Malarial Attacks.**—Dr. Loysel recommends the following formula for hypodermic injection in malarial attacks:

℞. Liq. ars nicalis ............................. *m* l,

Quiniæ sulph ............................. ʒ ss,

Tartaric acid ............................. q. s.,

Aqua destillata ............................. *m* clxl.

Injection for leucorrhœa—

Potassium chloratis ............................. grs. cc,

Vin. opii ............................. ʒ iij,

Aqua picis (tar water) ............................. ℥ viij.

—*London Medical Record.*

**Prescription for Headache.**—Dujardin-Beaumetz recommends the following:

℞. Caffeine ............................. gr. iv,
Salicylate of sodium ............................. gr. iv,
Hydrochlorate of cocaine ............................. gr. iss,
Water ............................. f ℥ ij,
Syrup ............................. f ʒ vss.

M. Take the whole at one dose at the beginning of the attack.
—*Maryland Medical Journal.*

**Digitalis as a Diuretic and Laxative.**—Huchard, in the *Revue de Clin. et de Ther.* of April 12, 1888, gives the following formula for a laxative powder containing digitalis:

Potass. sulphat .............................
Potass. tartrat .............................
Potass. nitrat ............................. aa gr. lx,
Fol. digital. (pulv.) ............................. gr. xv.

In twenty powders. One powder three times daily.

